# Diagnosing Thyroid Lymphoma: Steroid Administration May Result in Rapid Improvement of Dyspnea : A Report of Two Cases

**DOI:** 10.5812/ijem.11463

**Published:** 2014-01-01

**Authors:** Oliver S Eng, Sebastian Lesniak, Tomer Davidov, Stanley Z Trooskin

**Affiliations:** 1 Department of Surgery, Rutgers-Robert Wood Johnson Medical School, New Brunswick, New Jersey, USA

**Keywords:** Thyroid, Lymphoma, Dyspnea, Steroids, Trachea, Diagnostic Tests

## Abstract

**Introduction:**

Thyroid nodules, whether benign or malignant, are slow growing masses. There are certain clinical situations where sudden rapid growth may occur and cause acute respiratory compromise secondary to tracheal compression.

**Case Presentation:**

Here we describe two patients with suddenly enlarging thyroid nodules, who developed acute respiratory compromise in the absence of tracheal compression. Their symptoms rapidly improved with administration of corticosteroids, and in subsequent workup, both were diagnosed as thyroid lymphoma.

**Conclusions:**

The potent effect of corticosteroids in the rapid improvement of respiratory compromise associated with thyroid lymphoma represents an important clinical finding. This opens the possibility for the favorable response to corticosteroid therapy to be regarded as a possible preliminary diagnostic tool for thyroid lymphoma in acute respiratory distress patients in the absence of tracheal compression. Subsequent retrospective studies are necessary to verify this hypothesis.

## 1. Introduction

Thyroid nodules may be present in as much as 65% of the population (1), with 5-10% of palpable nodules harboring malignancy (2). Goitrous nodules can grow at a rate as low as under 1 cm over 38 months (3). In addition, thyroid nodules containing the slow growing papillary thyroid carcinoma (accounting for 90% of thyroid cancer) have been shown to grow at a similar rate (3). However, there are certain clinical situations where sudden rapid thyroid nodule growth may occur. First, sudden growth may be related to a benign goitrous nodule which has developed spontaneous hemorrhage (4). Second, longstanding nodules that develop into anaplastic thyroid carcinoma are known to experience rapid tumor growth and may present with tumoral hemorrhage as well (5, 6). Finally, thyroid lymphomas are rapidly growing tumors that might suddenly enlarge and result in acute respiratory compromise, secondary to tracheal compression (7) or possibly due to lymphocytic infiltration and/or inflammatory response. Lymphomas are known to be responsive to corticosteroids, and, consequently, many lymphoma treatment regimens use steroids to help arrest lymphoma growth (8). We describe here two patients with thyroid nodules that suddenly enlarged, resulting in acute respiratory compromise, but in the absence of tracheal compression. In both patients, symptoms rapidly improved with the administration of steroids in the emergency room. The subsequent histological examination of procedural specimens established the diagnosis of thyroid lymphoma. We suggest that the rapid improvement in acute respiratory distress in the absence of tracheal compression after steroid administration in a patient with enlarging thyroid nodules should be regarded as an important clinical finding for establishing a diagnosis of thyroid lymphoma.

## 2. Case Presentation

### 2.1. Patient 1

**Figure 1.**
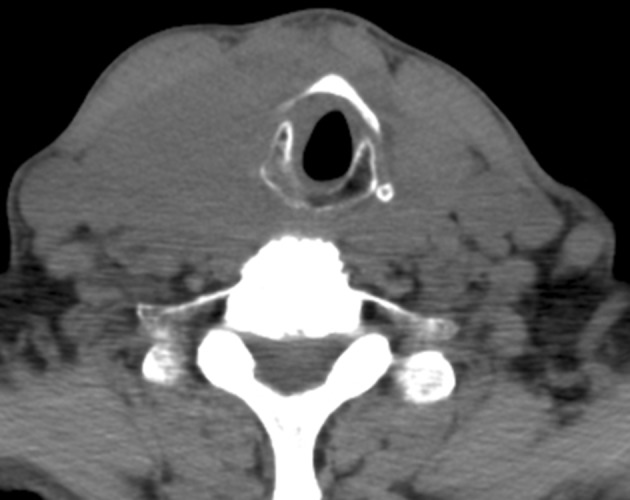
The CT Scan Showing Lack of Significant Tracheal Compression

A 78-year-old male was admitted with acute dyspnea and dysphagia for 3 days, and a 3-month history of increasing circumference of the neck. Two months prior to admission, ultrasound and computed tomography (CT) scan of the neck demonstrated two masses, of 6.8 cm and 5 cm each in largest diameter. Fine-needle aspiration (FNA) biopsies were consistent with Hashimoto’s thyroiditis, and the patient was subsequently started on levothyroxine. The patient’s past medical history included chronic atrial fibrillation, congestive heart failure, and chronic obstructive pulmonary disease (COPD). On presentation at admission, the patient’s vital signs were stable, with bilateral large and firm masses at cervical palpation, irregular heart rate, and bilaterally equal breath sounds. Given his symptoms, the patient was started on intravenous methylprednisolone. Cardiac workup and transthoracic echocardiogram showed a left ventricular ejection fraction of 65%. The cervical CT scan at admission, seen in Figure 1, revealed the enlargement of the thyroid into the anterior mediastinum, displacement of the trachea, but no evidence of tracheal compression. Flexible laryngoscopy evaluation revealed no upper airway distortion. The neck masses began to rapidly decrease in size, with concomitant symptomatic improvement with steroid administration. Repeat FNA indicated atypical lymphoid infiltrate arising in the setting of Hashimoto’s thyroiditis. Flow cytometry revealed lymphoid B-cells positive for CD19, CD20, Kappa+/CD19+, and Lambda+/CD19+, and negative for CD10. The polymerase chain reaction (PCR) study of immunoglobulin heavy chain gene rearrangement showed clonal B-cells with polyclonal background. To fully characterize the tumor, the patient then underwent open biopsy with pyramidal lobe and isthmus excision. Histological analysis of the specimen revealed diffuse large B-cell lymphoma positive for CD19, CD20, and monoclonal kappa light chains, but negative for CD5 and CD10, in the presence of thyroiditis. 

### 2.2. Patient 2

**Figure 2.**
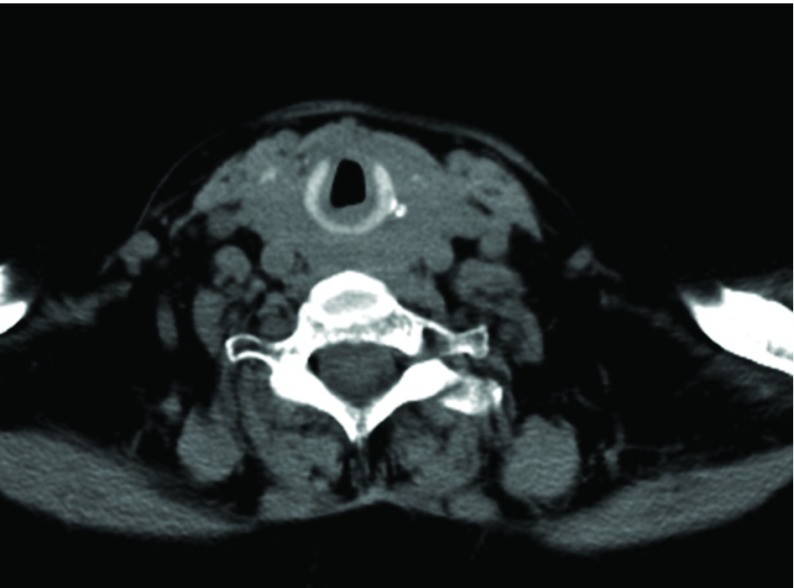
The CT Scan Showing the Absence of Significant Tracheal Compression

A 77-year-old female with a known hypothyroid goiter on levothyroxine therapy presented to the emergency room with a three-month history of dyspnea and intermittent stridor. The patient had no known history of asthma, COPD, or other cardiovascular or lung disease. Prior to admission, an extensive pulmonary evaluation had been performed, including pulmonary function tests, a ventilation-perfusion (V-Q) scan, and a laryngoscopy exam, all of which revealed normal findings. The patient had been started on oral prednisone 50 mg daily as an outpatient, and the symptoms of dyspnea improved significantly. However, as the dose was tapered, she experienced significant recurrence of dyspnea and cough. On presentation in the emergency room, bilateral large and firm cervical masses were palpated, while cardiovascular and lung examinations showed normal findings. Intravenous methylprednisolone was then administered, and the patient’s symptoms rapidly improved. The CT scan of the cervical region, seen in Figure 2, revealed diffuse heterogeneous enlargement of the thyroid gland without any evidence of airway caliber reduction from tracheal compression. The patient then underwent thyroidectomy. The histological analysis showed the specimen to be non-Hodgkin’s lymphoma, with diffuse large B-cell type positive for CD20, positive for BCL-2, negative for CD10, and predominantly negative for MUM-1.

## 3. Conclusions

Thyroid nodules, whether benign or malignant, are slow-growing (can be less than 1 cm over 38 months) (3). Acute respiratory compromise secondary to the sudden enlargement of thyroid nodules represents a rare, yet concomitantly life-threatening clinical entity. Spontaneous hemorrhage resulting in acute respiratory compromise from tracheal compression has been reported in the literature (4). Recently, a case report described a patient diagnosed with thyroid lymphoma after presenting to the emergency department with progressive respiratory distress from tracheal compression (7). In this case, the patient experienced respiratory arrest in the emergency department, was intubated, and underwent thyroid lobectomy and tracheostomy placement prior to steroid administration. The diagnosis of thyroid lymphoma was made after the histological examination of the surgical specimen (7). 

Thyroid lymphoma is an uncommon malignancy, comprising 2 to 8% of all thyroid malignancies (9). This disease often presents later in life and more commonly in women, with a recent study reporting a median age of 56 years, and a 58% female cohort (9). Dyspnea, stridor, dysphagia, and hoarseness are common symptoms in patients with thyroid lymphoma, with over 87% of patients presenting with an enlarging cervical mass (8, 9). Diagnosis is often established by combining elements from the patient’s history/physical exam, FNA, open biopsy, and/or surgical intervention. However, the utility of FNA is questionable. In a recent retrospective analysis of 64 thyroid lymphoma patients at a tertiary care center, only 28% of patients who underwent FNA during workup were pointed towards the diagnosis of lymphoma, and all of those patients then underwent a confirmatory open biopsy (9). Histological and phenotypic analyses such as flow cytology and immunohistochemistry have undergone significant improvements in diagnostic capability, and in the context of the major variability in the effectiveness of FNA in obtaining sufficient samples for testing, other diagnostic aids would be useful. Our findings in this report suggest one such potential aid. Most thyroid lymphomas derive from a B-cell origin, with diffuse large-cell type as the most common. Current treatment modalities include a combination of thyroidectomy, radiation, and R-CHOP (rituximab-cyclophosphamide, hydroxydaunorubicin, vincristine, prednisone) chemotherapy regimen (8). Treatment with multiple modalities (surgery, chemotherapy, radiation) has been shown to be essential, leading to an overall improved survival compared to single modality treatments (9).

The present paper reports the first known cases in which patients with enlarging thyroid nodules, ultimately diagnosed with thyroid lymphoma, experienced acute respiratory compromise in the absence of tracheal compression. Symptoms rapidly improved with prompt steroid administration, and these cases may represent an important clinical finding to aid in the diagnosis of thyroid lymphoma. This hypothesis should be investigated in future large scale retrospective studies.
